# Nurses’ Caring Experiences of Older Adults Who Attempted Suicide in Intensive Care Units

**DOI:** 10.1192/j.eurpsy.2026.10676

**Published:** 2026-07-31

**Authors:** Y.-F. Tsai

**Affiliations:** School of Nursing, Chang Gung University, Tao-Yuan , Taiwan

## Abstract

**Introduction:**

Elder suicide is a major global health concern. Despite its significance, limited research has examined the experiences of Intensive Care Unit (ICU) nurses in caring for older adults who attempted suicide.

**Objectives:**

This study aimed to explore ICU nurses’ caring experiences with older adults who attempted suicide.

**Methods:**

A qualitative descriptive approach was adopted. Eight ICU nurses from a medical center were recruited. Data were gathered through face-to-face, in-depth individual interviews using a semi-structured guide and analyzed using content analysis.

**Results:**

Participants reported that wrist or neck cutting, hanging, and ingestion of toxic substances (e.g., sulfuric acid) were common suicide methods among their older patients. Nurses described feeling shocked and unprepared, often questioning the patients’ motives at the time of the event. They also reflected on whether anything could have been done to prevent the attempt. Only two participants had sought professional support, such as consulting social workers to explore patients’ support systems or psychiatrists to assess mental health conditions.

**Conclusions:**

Caring for older adults who have attempted suicide is a recurring challenge in ICUs. However, nurses may lack adequate support and resources to manage these challenging situations. Targeted nursing education focusing on early detection and prevention of suicide in older adults is essential to strengthen ICU nursing practice.

**Disclosure of Interest:**

None Declared

